# Cultural landscape perception of the Chinese traditional settlement: Based on tourists’ online comments

**DOI:** 10.1371/journal.pone.0283335

**Published:** 2023-04-13

**Authors:** Jingjing Zhou, Shanshan Wu, Xiaojing Wu, Xiumin Xia

**Affiliations:** 1 College of Design, Ningbo Tech University, Ningbo, Zhejiang, China; 2 College of Architecture and Urban Planning, Guizhou University, Guiyang, Guizhou, China; Northeastern University (Shenyang China), CHINA

## Abstract

The cultural landscape in traditional settlements is an important historical and cultural resource created by human beings in the process of historical evolution, and is an important resource for the development of traditional settlement tourism. This paper selected 21 representative traditional settlements for research using online comments from tourists as data and content analytical methods including high-frequency vocabulary, semantic networks and emotional attitudes to explore the public perspective on the connotations of cultural landscape features in traditional settlements. There are four major findings, showing first that the cultural landscape of traditional settlements contains three core elements. Second, the semantic network relationships of the core elements show a significant central–edge tendency; and third, the emotional perception of cultural landscapes in traditional settlements is generally positive, and there is no significant difference emotionally between each core element. Last, the public’s perception of the cultural connotations of the landscape is seriously insufficient. Based on the research results, planning suggestions and countermeasures for the conservation and utilization of cultural landscapes in traditional settlements are proposed.

## 1 Introduction

Traditional settlements have experienced a long historical evolution and contain rich historical information and cultural resources. The important cultural landscape created by human beings in the course of their historical evolution is an important subject of study [1]. According to the classification of cultural landscapes in the “Operational Guidelines for the Implementation of Convention Concerning the Protection of the World Cultural and Natural Heritage”, traditional settlements belong to the continuation category under the classification of organically evolving landscapes. A cultural settlement landscape is defined as a group of historical buildings, structures together with the surrounding environment and the spontaneous growth of the building community. The settlement landscape continues social functions and shows the evolution and development of history [2]. It is an important relic of traditional culture, an invaluable measure of heritage left to the contemporary generation by our ancestors, and a nonrenewable cultural resource with high historical, cultural, scientific and artistic values.

With the rapid advance of industrialization and urbanization, traditional settlements that rely on traditional lifestyles and methods of production have gradually lost their foundation for survival and encountered difficulties in development [3]. The decline in traditional settlements is increasingly aggravated [4], which raises the prospects of the destruction of material culture such as architecture and living environments [5], the decline in the intangible culture of villages [6,7], and the conflict between the protection and utilization of the cultural landscape [8,9]. As one of the primary forms of protection for living heritage, tourism development has become a way to reverse the declining fate of many traditional settlements [10–13]. In the development of the cultural tourism of traditional settlements, the cultural landscape has become an important resource [14,15]. When the cultural landscape of traditional settlements plays a leading role in cultural tourism development, the landscape can effectively lead and promote the protection and rebirth of traditional settlements [16].

Most of the studies on traditional settlement cultural landscapes have been limited to the scope of cultural geography. The contents of these studies have included the characteristics, historical evolution and formation mechanism of cultural landscapes. However, with the development of traditional settlement tourism, the cultural landscape has become an important tourism resource and an important planning object in traditional settlement tourism planning. Therefore, it is necessary to study the traditional settlement cultural landscape from the interdisciplinary perspective of tourism, communication and planning. Against this research background, this paper focuses on the analysis of the types, characteristics and cultural connotations of the traditional settlement cultural landscape from the perspective of tourists. The research conclusion reveals the multi-dimensional characteristics and relationship of traditional settlement cultural landscapes from the perspective of tourists and provides decision-making suggestions for the protection and utilization of traditional settlement cultural landscape [17].

Based on the above research motivation, in terms of research methods, this paper uses online comments left by tourists as research data. The data come from the subjective evaluations of tourists and objectively reflect the cultural landscape characteristics perceived by tourists. Through preliminary data processing, the comments related to cultural landscape are extracted for content analysis, including high-frequency vocabulary, semantic network and emotional tendencies.

## 2 Review

### 2.1 Research on perceptions of traditional settlement cultural landscapes

According to the landscape intertextuality theory of new cultural geography, any scholar’s understanding of cultural landscape cannot be completely consistent with the meaning of the builder [18]. Cultural landscape perception continues the research paradigm and methods of landscape perception, widely uses psychological methods, and focuses on the use of empirical research methods such as experiments and tests to collect quantitative data [19–21]. The results have strong objectivity and operability [22]. According to the empirical research on cultural landscape perception, landscapes can be divided into scenes, including cultural landscape real scene perception, cultural landscape photo perception and cultural landscape 3D virtual scene perception. Cultural landscape reality and cultural landscape photos are common perceptual scene settings [23,24]. In recent years, with the development of SD and VR technology, many scholars have replaced landscape reality with virtual scenes. Compared with landscape reality, photos and virtual scenes have the advantages for researchers of easy measurements of eye movements, easy access to data, and accurate screening of samples of perceptual objects and subjects [25]. However, in perceiving photos and VR virtual scenes, except for the movement of the eyes, the perceiver does not actually produce physical movement with other parts of the body. Embodied cognition theory suggests that there is a strong connection between human physiological experience and psychological state [26,27]. People’s perceptual ability, such as the breadth, threshold and limit of perception, is determined by the physical properties of the body. In addition, people use all five senses to receive information from the landscape environment, while photos and virtual scenes cannot mobilize all of these five senses. Therefore, the difference between the perception of photos and VR scenes and the perception of landscape reality is obvious.

The data acquisition methods for empirical research on cultural landscape perception include questionnaire interviews [28,29], cognitive maps [30,31] and network data mining [32]. In the questionnaire and interview surveys, the data were collected and collated using the SD semantic difference method, Q method, metaphor extraction technology and other quantitative and qualitative methods. During data analysis, mathematical statistical tools such as principal component analysis, factor analysis, cluster analysis, multiple regression and structural equation modeling (SEM) are used for quantitative research [33].

In terms of research results on the perception of the cultural landscape of the traditional settlement, Daugstad started with the rural cultural landscape in Norway and investigated three-dimensional coordination related to the landscape: values and requirements, the method of experience, and future development prospects. The research shows that although participants hold different positions and attitudes, they all unite to pay attention to landscape changes and hope to protect traditional food and local agricultural products [34]. Stephenson believed that the information provided by recent planning theory and practice gave less attention to the significance and value of the cultural landscape. This research aims to develop a conceptual framework that integrates landscape planning elements and the perception of community cultural values to help understand the multicultural value of landscapes [35]. Li used GIS spatial analysis and participatory mapping to discuss the differences in type and spatial characteristics in resident perception of traditional village landscape values [36]. Chen used expert evaluation and public evaluation to conduct perception and preference studies on traditional village landscapes [37].

With the development of traditional settlement tourism, tourists have become an important new subject in the study of cultural landscapes in traditional tourist settlements are perceived. The perception of the cultural landscape in traditional settlements by tourists, as well as the difference from perception by other subjects (local residents, local governments and investors), has become a hot topic for scholars to study. Beeho conducted an ASEB semigrid analysis of tourists’ experience in New Lanark Village, a world cultural heritage site in the United Kingdom, by using in-depth interviews with tourists and found that the main experience of tourists is to enjoy historical and cultural education and benefit from learning [38]. Zeppel’s empirical analysis of an indigenous village in Canada found that tourists’ understanding of the history of traditional villages and contact with first nations were important factors affecting their cultural experience. It is proposed that the interpretation of indigenous culture should be key to improving the attraction of local indigenous villages. Traditional village tourism itself is driven by a certain motivation to seek difference, but different tourists pursue this experience of difference in different ways [39]. Lin studied the interaction between the perception of traditional village landscape contexts and tourist behavior [40]. Yang took the Longji Terrace in Guangxi as a case study, using literature analysis, online text analysis, rooting theory, etc., to study tourists’ perceptions through high-frequency vocabulary, perception category construction, emotion analysis, etc., and discussed tourists’ perception of the Longji Terrace agricultural cultural landscape, landscape perception differences, and insufficient tourism development, etc. [41]. Yuan tried to collect feedback on tourists’ visual perception of the cultural landscape of the ancient city of Pingyao by means of visual information extraction and other methods and used color geography, color psychology and other related theories and methods to extract and screen the characteristic visual elements of the city, providing visual references for subsequent planning and construction [42].

In summary, the research on the perception of traditional settlement landscapes mainly focuses on the difference and evaluation of the perception of different subjects to enhance local identity and the attachment of local residents, improve the satisfaction with traditional settlement tourism, and guide the planning, design and construction of the cultural landscapes of traditional settlements.

### 2.2 Study on the constituent elements of the cultural landscapes of traditional settlements

According to the combination of materiality and immateriality of cultural landscapes and the characteristics of traditional settlements, scholars have analyzed the cultural landscape of traditional settlements and its constituent elements (Table 1).

**Table 1:** Cultural landscapes of traditional settlements and constituent elements.

Scholars	Constituent Type	Constituent Elements
Zhao [43]	**Material landscape** **Nonmaterial landscape**	**·Material landscape:** buildings, roads, farmland infrastructure, water conservancy facilities, rural industry, agricultural production, crops, and rural residents **·Nonmaterial landscape:** rural lifestyle, rural production relations, rural environment, rural life, rural morality, rural aesthetics, rural wealth, rural customs, religious beliefs, rural production concept
Li [2]	**Material system** **Value system**	**·Material system:** Behavior: daily behavior, festival ceremony, traditional skills Architecture: historical architecture, local architecture Space: space composition, space form Structure: spatial structure, landscape structure Environment: landscape environment, pastoral scenery, green vegetation **·Value system:** Habitat culture: local customs, life philosophy, life culture Historical culture: historical evolution, historical events, historical figures Industrial culture: industrial history, industrial development, industrial characteristics Spiritual culture: cultural concept, aesthetic taste, spiritual belief
Liu [44]	**Landscape features** **Cultural features**	**·Landscape features:** Residential characteristics: quadrangle, tulou, dry fence, etc. Totem sign Main public buildings: ancestral temple, drum-tower, stone arch, bridge, etc. Environmental factors: big banyan trees, water network, mountains, lakes, rivers, etc. **·Cultural features:** Cultural features implied in ancient totems Cultural features implied in folk art Cultural features implicit in the traditional architectural landscape
Li [45]	**Natural environment** **Spatial form** **Residential type** **Social culture**	**·Natural environment:** landform, elevation, river grade, river relationship; ·**Spatial form:** settlement scale, settlement orientation, settlement form, street pattern; **·Residential type:** residential type system, building materials, façade characteristics, decorative style, typical buildings; **·Social and cultural aspects:** ethnicity (language/dialect), intangible cultural heritage
Long [46]	**People** **Events** **Places** **Things**	**·People** Elements: creator, owner, manager, user Value connotation: social value and spiritual value **·Events** Elements: functional behavior, ritual behavior, social behavior Value connotation: economic value, technological value, spiritual value **·Places** Elements: material attributes, spiritual attributes, institutional attributes Value connotations: ecological value and aesthetic value **·Things** Elements: fixed landscape elements, semifixed landscape elements, and nonfixed landscape elements Value connotation: historical value, aesthetic value, spiritual value
Li [47]	**Natural background** **Physical environment** **Mental world**	**·Natural background:** the human land relationship of scene geography **·Physical environment:** the spatial relationship between people and architecture **·Mental world:** cultural interpretation of spirit anthropology

### 2.3 Research on tourist perceptions based on web text

In the era of the network economy, various online media interaction platforms have been continuously used in the tourism field and have gained greater attention [48]. Tourism experience is often communicated and disseminated on these platforms [49]. Tourists can freely express and release their views, including travel notes, comments, strategies and other media on the internet [50,51]. Tourists explore and publicize their destinations in a "decentralized" way. Therefore, tourist perceptions of destinations no longer depend on traditional propaganda but on user-generated content (UGC), such as tourism virtual communities and media-related tourism [52,53]. The content of "decentralized" UGC includes not only words but also pictures, videos and other media forms, which are richer, more vivid and more realistic to reflect the tourism experience at the destination [54]. In recent years, scholars have made full use of social media data and emerging online textual analysis methods to study the perceived value and quality of tourism and its elements, structure and influencing factors [55,56]. This method can effectively avoid the shortcomings of collecting information through questionnaires and obtain more authentic ideas from tourists in their own words [57]. The research sample is collected online, which simplifies offline data processing procedures and lowers the threshold for researchers; qualitative research tools can also ensure the effectiveness of the research.

### 2.4 Review summary

According to the landscape intertextuality theory from the New Cultural Geography, different groups have different understandings of cultural landscapes, so it is necessary to study cultural landscape perception from the perspective of tourists to improve traditional settlement tourism planning. Cultural landscape perception is based on the research paradigm and methods of landscape perception, and the empirical research methods of psychology are widely used. With the development of VR technology, more empirical studies have constructed virtual scenes, but there are still differences between physical perception based on real scenes and virtual scene perception. Realistic perception research has certain advantages.

The purposes of the literature on cultural landscape perception are to explore spatial differences among cultural landscapes, differences in group perception, multi-dimensional significance and values, and factors affecting cultural landscape perception. In terms of research methods, social media data and online text analysis are widely used. These methods provide a methodological reference for this paper. In addition, a large number of research results are based on the types and characteristics of traditional village cultural landscapes from the perspective of cultural geography scholars in previous studies, and these results are the basis of the perception research presented in this paper.

## 3 Methods

### 3.1 Research data

#### 3.1.1 Research object

According to the official evaluation standard in China, traditional settlements include famous historical and cultural towns and villages in China as well as traditional villages. The evaluation of famous historical and cultural towns and villages in China began in 2003. The evaluation criteria include those towns and villages with rich cultural relics and great historical value or commemorative significance, which can fully reflect the traditional style and features of some historical periods or local ethnic characteristics. To date, seven batches have been selected, amounting to 312 famous historical and cultural towns and 487 famous historical and cultural villages [58]. The evaluation of traditional villages began in 2012. The evaluation criteria include those villages that have an early history, rich traditional resources, and certain historical, cultural, scientific, artistic, social, and economic values and should be protected. To date, five batches have been selected, amounting to 6823 traditional villages [59].

#### 3.1.2 Data source

The popularity of the Internet makes it more convenient for tourists to record scenic spots, itineraries and feelings after visiting a location. "Online comments" can directly record the characteristics of tourist stops and describe the observations and feelings that occurred in response. The content related to cultural landscape perception is selected from the online comments from traditional settlement tourists as research data. This data acquisition method has the advantages of authenticity, universality, and accessibility, etc., and is reasoned and feasible. The online comment text is mainly from UGC-type websites. The daily ranking of domestic tourism websites is obtained through querying the Alexa ranking website, and Ctrip (www.ctrip.com), which ranks first, is selected as the source of comment data. No specific permits were required for getting all the online comments data, as all the data from the website of Ctrip (www.ctrip.com) did not involve human participants, human specimens or tissue, vertebrate animals or cephalopods, vertebrate embryos or tissues or field research. Further, there is no conflict of interest in the choice of data source, as all data comes from a public website (www.ctrip.com) and is freely available to the public.

#### 3.1.3 Research sample

Considering the efficiency and scientific nature of the research and focusing on the richness of the research sample, this study tries to screen samples that can provide high-density information for the research. This study follows the following principles in selecting research samples of traditional settlements: (1) typicality: qualifying traditional settlements are required to be typical of regional, geographical and cultural characteristics [60,61]. (2) Extensiveness: the selected samples cover the different types of traditional settlements in China as much as possible. (3) Nonspecific: the selected sample should be able to represent a certain type of settlement, rather than isolated examples. Considering the particularity of ethnic minority settlement culture, it is not included in the screening scope. (4) Nonoverlapping: only one sample is selected for traditional settlements of the same type. There are differences among samples, providing as much information as possible for the study. (5) Feasibility: The selected samples must be well represented in web-published comments, which can reflect enough common characteristics for researchers to analyze the content.

Based on the above 5 principles of sample selection and the number of comments on the Ctrip website, villages under the same circumstances and with more comments are selected to meet the needs of research and analysis. Finally, 21 traditional villages were selected from 6,823 research objects as the research sample (Table 2). This research sample represents the typical types of traditional Chinese villages, and the dataset contains enough comments on the Ctrip website to support data analysis.

**Table 2:** Research sample list.

No.	Name	Province	Geographical Environment	Cultural Characteristics	Comments
1	Cuandixia	Beijing	Central part of the canyon	Traditional residence	316
2	Yangliuqing	Tianjin	Plain	Folk culture and art	320
3	Jimingyi	Hebei	Plain	Historic Posthouse	671
4	Zhangbi	Shanxi	The Loess Plateau	Pocket military castle	854
5	Qinglong	Shanxi	Plain	Military Town	299
6	Luxiang	Jiangsu	Back to the mountain and facing the water	Hall Buildings in Ming and Qing Dynasties	607
7	Nianbadu	Zhejiang	Mountainous region	Cultural enclave	1032
8	Xinye	Zhejiang	Back to the mountain and facing the field	Clan culture	428
9	Tangmo	Anhui	Water reticulation plain	Shuikou Garden	624
10	Peitian	Fujian	Back to the mountain and facing the water	Hakka residential buildings	434
11	Huangling	Jiangxi	Mountainous region	Mountain Village	3429
12	Zhuquan	Shandong	Back to the mountain and facing the water	Ecological Ancient Village	2403
13	Shedian	Henan	Plain	Water land transit hub fortress	235
14	Dayuwan	Hubei	Valley plain	Traditional residence	267
15	Zhangguying	Hunan	Valley basin	Community of ancient residential buildings	345
16	Nanshe	Guangdong	Region of rivers and lakes	Guangfu Agricultural Settlement in Lingnan	488
17	Huangyao	Guangxi	Karst plain	Couplet culture	2923
18	Yangmei	Guangxi	Facing water	Ancient Commercial Town	202
19	Shangli	Sichuan	Back to the mountain and facing the water	Ancient road	420
20	Bingan	Guizhou	Mountainous region	Military and Commercial town	97
21	Qingmuchuan	Shanxi	River valley	Border Trade Town	454
Total					16848

Note: The comment data are from January 2015 to February 2022.

#### 3.1.4 Data collection

With the help of the free web crawling software "Octopus Collector", we collected tourist comment data about the 21 villages in the research sample on Ctrip (www.ctrip.com). Since some traditional settlements have few comments on Ctrip before 2014, only qualified data submitted in or after January 2015 is included to unify the time period across all data. Since the data was collected for this article in March 2022, the latest comments date to February 2022. A total of 16848 comments were collected, and the text was preprocessed. Researchers quickly read all comments one by one, conducted preliminary screening, and deleted comments that did not conform to the research content. The following three types of comments are mainly deleted: pure scenic spot information; pure tourism strategies; and empty or overly simple descriptions. After preprocessing the comments, 3426 comments were deleted, and the remaining 13422 comments were used for data analysis.

### 3.2Analysis methods

#### 3.2.1 Vocabulary segmentation and vocabulary frequency statistics

ROSTCM6 software was used to conduct vocabulary segmentation and vocabulary frequency statistics on the massive database of tourist online comment texts about traditional settlements, high-frequency vocabulary words related to cultural landscapes were selected, relevant words and concepts such as the composition, connotation and value of cultural landscape of traditional settlements were identified if also mentioned in the relevant literature [62,63], and the core element system was built for the perceptions of cultural landscapes in traditional settlements. This work laid a foundation for further analysis of semantic network structure.

#### 3.2.2 Semantic network analysis

Through the statistical analysis of vocabulary segmentation and vocabulary frequency, we can determine the main features of the traditional settlement cultural landscape that tourists pay attention to and the cultural connotations and value that tourists perceive, but we do not know the relationship and relevance of these words to the perception process. This paper uses semantic network analysis to analyze the structural relationship of the core elements of cultural landscape perception [64]. A semantic network is a structure network diagram expressed by concepts and semantic relations. It consists of nodes and connections between nodes [65]. Nodes represent things, concepts, attributes, operations, states, etc., while lines represent semantic connections between connected nodes [66]. This paper constructs the semantic network of text according to the frequency of co-occurring words.

#### 3.2.3 Analysis of emotional tendency

Emotional attitude analysis mainly analyzes the emotional nouns and adjectives used in the sample text and draws conclusions about the emotional tendency and perceptual attitude of tourists toward the cultural landscape of the traditional settlement. As an opinion, emotional tendency reflects tourists’ perceived level of satisfaction with the cultural landscape in the traditional settlement. Emotional tendencies can be divided into positive and negative [67]. This paper first analyzes the overall emotional tendency of network text and further takes core elements of perception as key words to analyze the emotional tendency in this specific subset.

### 3.3 Research procedures

The specific research procedures of this paper are as follows: (1) With ROSTCM6 software, 13422 texts after preprocessing are segmented to extract high-frequency vocabulary related to the perception of the cultural landscape in the traditional settlement; (2) With reference to the relevant literature, the extracted high-frequency vocabulary of cultural landscape perception is used to construct a table of categories of perceptual elements in traditional settlements. (3) By using network analysis, the semantic network structure is constructed for the core elements of language used to describe perceptions of traditional settlement cultural landscapes; (4) ROST EA 1.9.0.4 is used to analyze the overall emotional tendency of tourists’ perceptions of traditional settlement cultural landscape, as well as the emotional tendency around the core elements of perception. (5) According to the research results, this paper further analyzes the public perception of characteristics of the traditional settlement cultural landscape, public demands for the traditional landscape cultural landscape, and current shortcomings and puts forward countermeasures and suggestions for cultural landscape planning in traditional settlements.

## 4. Results

### 4.1 High-frequency vocabulary of traditional settlements cultural landscape perception

Through vocabulary segmentation and vocabulary frequency statistics on the online comment texts about traditional settlements, the high-frequency words describing characteristics of traditional settlement cultural landscapes are extracted (Table 3). The word-phrase “airing out farm goods in the autumn sun (shaiqiu)” has the highest frequency, reflecting that tourists have a strong perception of both visual aesthetic and agricultural characteristics in the cultural landscape. Second, there are words related to ornamental agricultural landscapes such as "canola flowers", "terrace" and "willow". In addition, tourists also have a strong perception of the architecture of cultural landscapes such as historical buildings and structures such as "ramparts", "ancestral temples" and "memorial archways". Tourists have the highest perception of traditional settlement culture with words such as "original ecology" and "simple folk customs", reflecting the ecological culture and local culture of the settlement. The characteristic words with the highest frequency reflecting the degree of perception of the cultural value of traditional settlements include "fun", "leisure", "experience" and "feeling", which reflect the economic and educational value of tourism.

**Table 3:** High-frequency vocabulary in traditional settlement culture perception.

Ranking	High-Frequency vocabulary	Frequency	Ranking	High-Frequency vocabulary	Frequency
1	Airing farm goods in the autumn sun	1121	26	Stilted building	168
2	Interesting	816	27	Memorial archways	164
3	Experience	776	28	Huizhou	157
4	Canola flowers	769	29	Original ecology	152
5	Feel	568	30	Vacation	148
6	Quiet	476	31	New Year pictures	147
7	Natural	471	32	Secluded	143
8	Fascinating	457	33	Simple and honest local people	140
9	Terrace	436	34	Typical	139
10	Leisure	429	35	Flagstone pavement	136
11	Bamboo forest	376	36	Entertainment	134
12	Willow	364	37	Brook	128
13	Anhui style	335	38	Spectacular	121
14	Historical culture	307	39	Quadrangle dwellings	106
15	Enthusiasm	280	40	Creek	106
16	Peaceful	268	41	Delightfully cool	104
17	Rampart	255	42	Water-bound country	103
18	Pure and fresh	243	43	Vicissitudes	103
19	Ancestral temple	197	44	Cultural deposits	102
20	Flowing water	197	45	Pleasantly cool	101
21	Unique	191	46	Well-proportioned	98
22	Carefree	185	47	Hakka	94
23	Enjoy	183	48	Fresh	94
24	A stream in front and a hill at the back	178	49	Canal	94
25	Ancient tree	173	50	Ancient well	93

Note: Due to limited space, only the top 50 high-frequency words are listed.

### 4.2 Element system of cultural landscape perception in traditional settlements

On the basis of extracting high-frequency vocabulary, combined with relevant literature on traditional settlement cultural landscape research, high-frequency words related to traditional settlement cultural landscapes in online texts are classified into 47 subcategories and further grouped into three categories related to cultural landscapes: entity, connotation, and value. There are 36 subcategories under the category of cultural landscape entities, covering buildings, built environment, natural environment, customs, daily behaviors and historical texts. The connotations of the cultural landscape includes four subcategories: clan culture, human settlement culture, farming-reading culture and rural culture. The value of cultural landscape covers seven subcategories: historical value, ecological value, spiritual value, artistic value, research value, educational value and economic value (Table 4).

**Table 4:** Element system of cultural landscape perception in traditional settlements.

Categories	Subcategories	High-Frequency Vocabulary
**Cultural landscape entity**	Construction	memorial archways, decorated archway, gatehouse, stele, glazed stele, rampart, stage, ancient bridge, stone bridge, covered bridge, drawbridge, a screen wall facing the gate of a house, screen wall, gazebo, long corridor, pavilion, waterwheel, stone mill
	Geomantic architecture	wenfeng tower, wenchang pavilion
	Ritual architecture	ancestral hall, ancestral temple
	Religious architecture	ancient temple, temple
	Educational building	school
	Former residence	senior official residence, Huihe Hall, Prime Minister’s house, Site of Guangxi Provincial Working Committee
	Commercial buildings	escort agency, Rong Shengkui, Shanshan Guild Hall
	Architectural space	courtyard, yard, atrium, hall, lobby, well shape, wing room, quadrangle dwellings
	Building structure	wood structure
	Architectural modeling	cornices, Ma Tau wall watts, steps, stone steps, ladder, stilted building, seats for beauties
	Architectural decoration	mural, color painting, pattern, stone carving, wood carving, carve patterns, engrave, enchase, brick carving, lantern, carved beams and painted rafters
	Building material	stone, gray brick, gray tile, green tile, colored glaze, loess, pink walls and black tiles, white wall and black tile
	Building technology	construction technology, exquisite technology and exquisite workmanship
	Architectural style	Hakka, Anhui style, Huizhou, Hui style, water-bound country, Lingnan style
	Open space	square
	Street pavement	flagstone pavement, cobblestone
	Street form	profound, long, looming in the distance, deep and serene, tortuous, meandering, winding
	Street structure	crisscross, complex and mixed, lead in all directions
	Street scale	narrow
	Architectural community	well-proportioned, row upon row of, tier upon tier, build along the mountain
	Space structure	fan-shaped, a stream in front and a hill at the back, well-formed
	Settlement atmosphere	quiet, peaceful, secluded, quiet and deep, tranquil, seclusion, calm
	Topographic features	surrounded by mountains, halfway up the mountain, on the cliff
	Air	pure and fresh, fresh, smoke from kitchen chimneys, delightfully cool, pleasantly cool, mist
	Water body	pool, pond, creek, brook, flowing water, waterway, well, ancient well, Zuo river, canal, water gap
	Greening vegetation	ancient tree, big banyan tree, big tree, camphor, maple, yew, willow, lotus, cherry blossom, gingko, osmanthus, moss, green trees, wild flowers, chrysanthemum
	Agroforestry fruit trees	terrace, farmland, canola flowers, chrysanthemum morifolium, peach blossom, loquat, pear flower, orange tree, bamboo forest, apricot tree
	Written record	horizontal inscribed board, plaque, antithetical couplet, couplet hung on the pillars, pedigree of a clan
	Handicrafts	carving, paper cutting, lanterns, New Year’s pictures, kites, wood carvings, leather-puppets, porcelain
	Commodity and Food	white jade square cake, gong cake, magnolia bud, walnut steamed bun, barrel fish, horseshoe powder, tofu flower, glutinous rice cake, snail powder, Huangyao fermented soybean, tofu, dada noodles
	Dialect idiom	dialect
	Daily life	washing vegetables, pickles, carefree, flavor of rural life
	Production activities	airing farm goods in the autumn sun
	Festival activities	March 3rd Festival, Lantern Fair, Temple Fair
	Folk customs	marriage custom, habitude
	Traditional skills	leather-puppet show, ancient drama, traditional opera, chicken fighting
**Cultural landscape connotation**	Clan culture	ancestor worship, clans, consanguinity, established practice, regulation
	Human settlement culture	geomantic omen, constellation, theory that man is an integral part of nature
	Farming-reading culture	cultural deposits, humanistic atmosphere, inspiring place producing outstanding and talented people
	Rural culture	Huizhou culture, local customs and practices, country flavor, simple and honest local people, simple and unadorned, good and honest, enthusiasm, hospitable, folk culture
**Cultural landscape value**	Historical value	historical culture, historical details, historical sense, heritage
	Ecological value	original ecology, ecology, natural
	Spiritual value	admiration, release of mood
	Artistic value	archaic rhyme, unpretentious, vicissitudes, elegant, refined, classical, spectacular, magnificent, grand, mysterious, local or native art or handicraft, classic works
	Research value	unique, distinctive, peculiar, rare, unique, matchless, save complete, typical, living fossil
	Educational value	learn, experience, feel, appreciate
	Economic value	fascinating, interesting, leisure, vacation, entertainment, enjoy

### 4.3 Semantic networks of traditional settlement cultural landscape perception

In this paper, the high-frequency words in the web texts are replaced by the corresponding subcategory names. This process summarizes how many scattered high-frequency words are placed into corresponding subcategories to clearly show the core network relationship.

**Fig 1:**
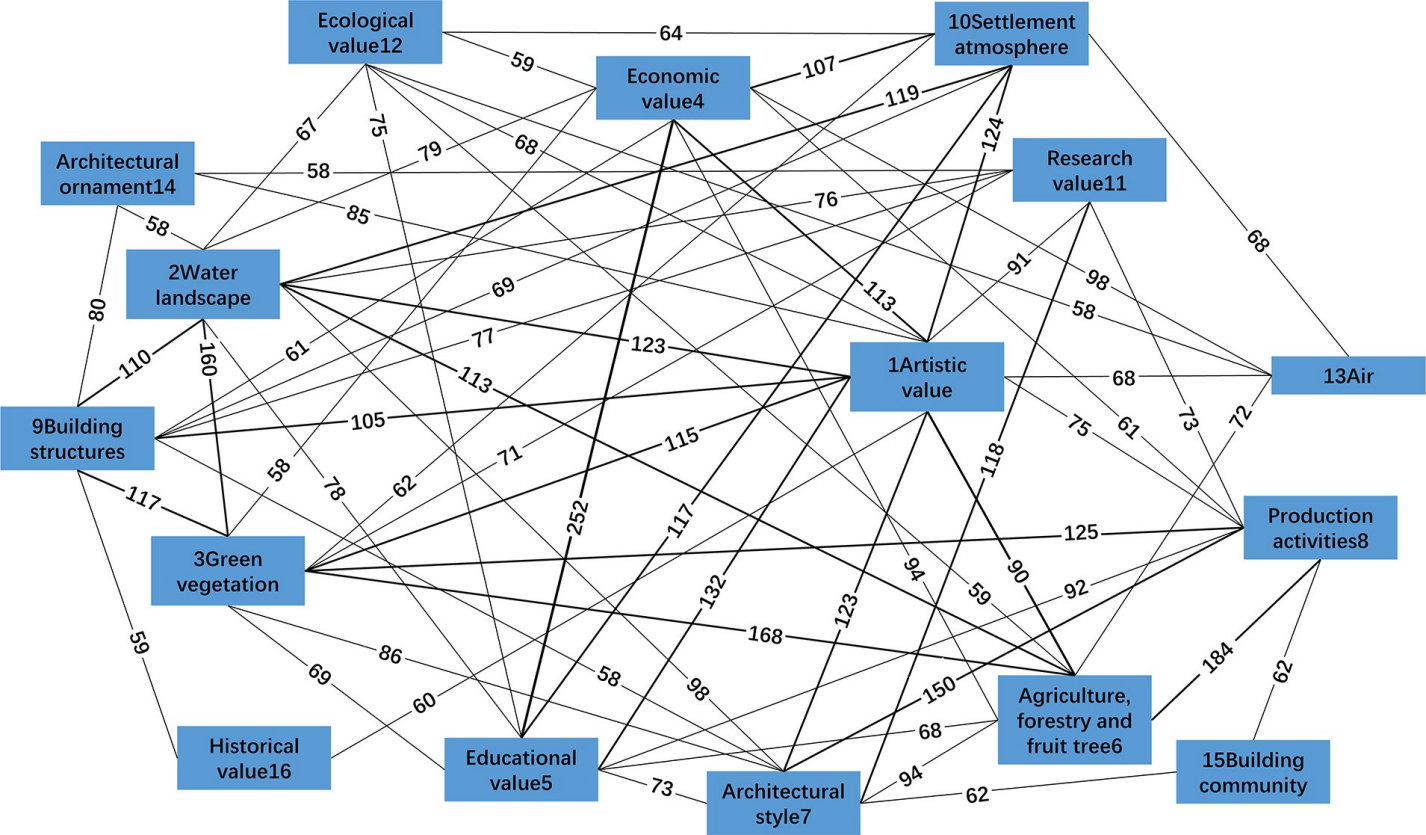
Semantic networks of traditional settlement cultural landscape perception.

As shown in Fig 1, the value perception system is composed of artistic value, economic value, ecological value, historical value, research value and educational value. The core cultural landscape entities supporting these value perceptions include water bodies, settlement atmosphere, various architectural constructions, green vegetation, agroforestry fruit trees, architectural styles, production activities and sub core cultural landscape entities, including architectural communities, air and architectural decoration.

In this network structure, the seven subcategories for the value of traditional settlement cultural landscapes, except for spiritual value, are included, indicating that the value of settlement cultural landscapes is generally recognized. However, none of the cultural connotations contained in the cultural landscape are included in this structure, indicating that the cultural connotation characteristics are not significant enough to be effectively considered and perceived. In terms of cultural landscape entities, only 10 subcategories are included, accounting for 27.8% of the 36 entity subcategories. There is an obvious core edge structure in cultural landscape perception.

### 4.4 Emotional tendencies in traditional settlement culture landscape perception

The overall emotional tendencies of tourists’ perception of the traditional settlement cultural landscape are analyzed by using the emotional analysis software ROST EA, as shown in Table 5.

**Table 5:** The general emotional tendencies in traditional settlement culture landscape perception.

Emotional Tendency	Positive Emotion	Neutral Emotion	Negative Emotion
Proportion	65.54%	0.73%	33.73%

Furthermore, 47 subcategories of traditional settlement cultural landscape perception were taken as keywords to analyze tourists’ emotional tendencies. Table 6 shows the analysis results. It can be seen from the analysis results that the terms “settlement atmosphere” and “economic value” (65.71%) with the highest positive emotion are only 0.17% different from the terms with the least positive emotion: “geomantic architecture”, “educational building”, “former residence”, “street scale”, “dialect idiom”, “traditional skills”, “clan culture” and “rural culture” (65.54%). The “settlement atmosphere” with the lowest negative emotion (33.56%) is only 0.17% different from the terms with the least negative emotion: “geomantic architecture”, “educational building”, “former residence”, “street pavement”, “street scale”, “dialect idiom”, “traditional skills”, “clan culture” and “rural culture” (33.73%). According to the analysis, the emotional tendencies across 47 subcategories of traditional settlement cultural landscape perception tend to converge significantly.

**Table 6:** The emotional tendencies of 47 perceptual subcategories of traditional settlement cultural landscapes.

47 Subcategories	Positive Emotion	Neutral Emotion	Negative Emotion
Settlement atmosphere	65.71%	0.73%	33.56%
Economic value	65.71%	0.71%	33.58%
Water body	65.67%	0.68%	33.65%
Artistic value	65.66%	0.72%	33.62%
Air	65.65%	0.72%	33.63%
Construction	65.63%	0.70%	33.67%
Production activities	65.63%	0.71%	33.66%
Educational value	65.62%	0.72%	33.66%
Architectural modeling	65.61%	0.69%	33.70%
Architectural style	65.61%	0.72%	33.67%
Green vegetation	65.61%	0.72%	33.67%
Architectural decoration	65.60%	0.73%	33.67%
Space structure	65.60%	0.70%	33.70%
Agroforestry fruit trees	65.60%	0.73%	33.67%
Historical value	65.60%	0.72%	33.68%
Ritual architecture	65.59%	0.73%	33.68%
Architectural space	65.59%	0.72%	33.69%
Building material	65.58%	0.70%	33.72%
Architectural community	65.58%	0.73%	33.69%
Daily life	65.58%	0.72%	33.70%
Farming-reading culture	65.58%	0.73%	33.69%
Research value	65.58%	0.73%	33.69%
Topographic features	65.57%	0.73%	33.70%
Commodity and Food	65.57%	0.73%	33.70%
Ecological value	65.57%	0.73%	33.70%
Religious architecture	65.56%	0.73%	33.71%
Commercial buildings	65.56%	0.73%	33.71%
Building technology	65.56%	0.73%	33.71%
Street form	65.56%	0.73%	33.71%
Handicrafts	65.56%	0.72%	33.72%
Human settlement culture	65.56%	0.73%	33.71%
Building structure	65.55%	0.73%	33.72%
Open space	65.55%	0.73%	33.72%
Street pavement	65.55%	0.72%	33.73%
Street structure	65.55%	0.73%	33.72%
Written record	65.55%	0.73%	33.72%
Festival activities	65.55%	0.73%	33.72%
Folk customs	65.55%	0.73%	33.72%
Spiritual value	65.55%	0.73%	33.72%
Geomantic architecture	65.54%	0.73%	33.73%
Educational building	65.54%	0.73%	33.73%
Former residence	65.54%	0.73%	33.73%
Street scale	65.54%	0.73%	33.73%
Dialect idiom	65.54%	0.73%	33.73%
Traditional skills	65.54%	0.73%	33.73%
Clan culture	65.54%	0.73%	33.73%
Rural culture	65.54%	0.73%	33.73%

## 5. Discussion

### 5.1 Structural relationships in the perceptions of traditional settlement cultural landscapes

The vocabulary frequency analysis shows that there is a long tail in the distribution of high-frequency words in the perception of traditional settlement cultural landscapes; that is, a few words are used very frequently, while most words are used very infrequently. The analysis of the long tail distribution of high-frequency words shows that the public very frequently perceives the cultural landscape to embody rural, historical and natural characteristics and visual aesthetic effects. A series of personalized, refined and knowledgeable cultural landscapes are distributed in the tail.

Further analysis of the semantic network shows that there is a significant core–edge structure in the perception of traditional settlement cultural landscapes; that is, there are core elements and edge elements in the perceptual elements of cultural landscapes. The perceptual frequency of core elements is high, and they are in the central position in the semantic network, closely related to other elements. In addition to the core elements, there are a large number of edge elements. Their perceptual frequency is low, and they are at the edge of the semantic relationship network or are not included in the network. Their relationship with other elements is weak, and the proportion of edge elements is much higher than that of the core elements. This indicates that tourists pay attention to and perceive only the specific and few cultural landscapes of traditional settlements and automatically ignore other cultural landscapes.

### 5.2 Emotional characteristics of perceptions of traditional settlement cultural landscapes

According to the analysis of emotional tendencies, the positive emotional tendency toward the cultural landscape is twice as frequent as the negative emotional tendency, and the neutral tendency accounts for a small proportion, indicating that the emotional tendency toward the cultural landscape of traditional settlements is generally positive, which indicates that the cultural landscape has played a positive role in the tourism development of traditional settlements.

Furthermore, cultural landscape perceptual elements are taken as the key words for emotional analysis. Research data show that tourists’ emotional tendencies toward various elements have significantly converged, indicating that emotional tendencies are not subject to changes in response to cultural landscape element types. The emotional tendency of tourists comes from the comprehensive perception of all cultural landscapes.

In terms of emotional tendency, there is no obvious difference between different types of cultural landscapes. Therefore, it can be inferred that tourists have no special preference for specific types of cultural landscapes. The emotional differences mainly come from the characteristics of tourists themselves and the quality of the cultural landscapes.

### 5.3 Progressive levels in the perceptions of traditional settlement cultural landscapes

Semantic analysis was used to explore the progression of perceptions of traditional settlement cultural landscapes. According to the semantic features of high-frequency words in cultural landscape descriptions, these words can be classified into those related each to cultural landscape entity, cultural landscape connotation and cultural landscape value. In cognitive psychology, different schools hold different or even opposing views on the activity and dynamic mechanism of human perception. But in general, the three-stage cognitive process of "feeling-perception-cognition" is widely recognized [68]. Referring to the above theories, this paper divides the chronological relationship of traditional settlement cultural landscape perception into three progressive levels: the perception of cultural landscape as an entity, the recognition of the connotations of the cultural landscape, and the judgment of the value of the cultural landscape.

The sensation of cultural landscape entities not only comes from vision but also includes elements of the cultural landscape acquired by hearing, taste, smell and other senses, such as dialect idioms, food, air, etc. The cultural connotation contained in these cultural landscape entities is interpreted and perceived by tourists to form a perception of cultural connotation, including clan culture, human settlement culture, farming culture and local culture, and further deduce and judge the value of cultural landscape, including historical value, ecological value, spiritual value, artistic value and other direct values, as well as research value, educational value and economic value based on development and utilization. The above three levels reveal the progressive process of perceiving traditional settlement cultural landscapes, which provides an effective analytical framework and research content for researchers to recognize the contours of the traditional settlement cultural landscape.

The perception of the value of the cultural landscape mainly focuses on the subjective value of the perceiver, such as economic and educational value, while the perception of its objective value is weak. In addition, although the cultural landscape resources are rich and the public has a good perception of cultural landscape entities, the public’s perception of the cultural connotations in the traditional settlement cultural landscape is very weak. However, the high degree of recognition of the value of the cultural landscape shows that traditional settlement culture has the potential for inheritance, innovation and development.

In general, the public does not understand the history and culture of the traditional settlement cultural landscape, so we should develop the "soft" content of the cultural landscape and conduct in-depth research and exploration on its cultural connotations and historical change. Publicize the background knowledge and cultural connotations of the traditional settlement cultural landscape and let the public know more about the cultural connotations of the cultural landscape through social media promotion and folk custom activity planning, etc. The public’s awareness of the cultural landscape of traditional settlements can be improved along with the public’s awareness of the need to protect traditional settlements and the public can be guided to participate in the protection and inheritance of "traditional settlements".

## 6. Conclusion

This study constructed the core elements of the cultural landscape from the perspective of the public and analyzed the network structure of the core semantic elements and the emotional tendencies based on online comments from tourists and related documents on traditional settlement cultural landscapes. This study makes two contributions. In terms of theory, it provides a framework and reference for further understanding the connotations and values of the cultural landscape of the traditional settlement from the perspective of the public and supports policy-makers seeking to protect and develop traditional settlements. In practice, it helps traditional settlement administrators understand the key assets and potential opportunities for the protection and utilization of cultural landscape resources.

In the planning of cultural landscapes, not only is the physical planning, or "hardware", important, so is the integration of cultural connotations, or "software", into the cultural landscapes. Sufficient historical and cultural background knowledge is a necessary condition to enhance the public’s sensitivity to cultural landscapes. On the basis of the presentation of cultural landscape entities, "software" such as historical and cultural background knowledge can be displayed and publicized. In addition to the traditionally static physical reality, virtual reality can also be used to organically present this combined programming of "software" and "hardware" of the cultural landscape. In cultural landscape planning, we should fully tap the resources embodied in the characteristics of traditional settlements, organize cultural landscapes with similar connotations into a series of connected landscapes, highlight their shared characteristics and themes, and enhance the perceptibility of cultural connotations. The spatial planning of the cultural landscape uses culture as the organizing principle for the design of the tour route through the landscape. A series of landscapes with clear cultural themes is a convenient means of public education to identify cultural connotations, providing a good foundation for the protection and inheritance of cultural landscapes.

However, this study also has some limitations. First, the research data do not cover tourists who do not use the internet, including some elderly tourists. Second, the data used in this study are mainly web text comments. In reality, many tourists use photos or videos to express themselves. We will enrich the information sources in subsequent research. Third, the cultural landscape of traditional settlements is a complex cultural construction. The quantitative analysis of big data alone cannot fully tap the cultural landscape resources and cultural connotation. Qualitative research methods (such as interviews) should be added to follow-up research to enhance the depth of the research into cultural landscapes. Finally, these data come from the web and do not involve basic demographic information, such as age and gender. In the future, it will be possible to investigate the differences and diversity of cultural landscape perception in combination with the demographic characteristics of tourists.

## Supporting information

S1 Dataset Original online comment data. (XLSX)

S2 Dataset Valid data after deleting invalid online comments. (XLSX)
